# The Protective Effects of Hydrogen Sulfide New Donor Methyl *S*-(4-Fluorobenzyl)-*N*-(3,4,5-Trimethoxybenzoyl)-l-Cysteinate on the Ischemic Stroke

**DOI:** 10.3390/molecules27051554

**Published:** 2022-02-25

**Authors:** Jing Fan, Junxi Du, Zhongwei Zhang, Wenjing Shi, Binyan Hu, Jiaqin Hu, Yan Xue, Haipeng Li, Wenjin Ji, Jian Zhuang, Pengcheng Lv, Kui Cheng, Kun Chen

**Affiliations:** 1 The Joint Research Center of Guangzhou University and Keele University for Gene Interference and Application, School of Life Science, Guangzhou University, Guangzhou 510006, China; jingfan002@gmail.com (J.F.); junxi4563@163.com (J.D.); shiwenj98@163.com (W.S.); hubn32@163.com (B.H.); jiaqinhuuu34@gmail.com (J.H.); lihaip25@163.com (H.L.); 2 Intensive Care Unit, West China Hospital, Sichuan University, Chengdu 610041, China; zhang643@sina.com; 3 Department of Cardiovascular Surgery, Guangdong Cardiovascular Institute, Guangdong Provincial People’s Hospital, Guangdong Academy of Medical Sciences, 96 DongChun Road, Guangzhou 510080, China; xueyhh2@gmail.com (Y.X.); wjima655@163.com (W.J.); zhuangji2753@163.com (J.Z.); 4 School of Pharmaceutical Sciences, Southern Medical University, Guangzhou 510515, China

**Keywords:** SAC and gallic acid conjugate, H_2_S new donor, MTC, ischemia reperfusion, neuro-ischemic drug candidate

## Abstract

In this paper, we report the design, synthesis and biological evaluation of a novel *S*-allyl-l-cysteine (SAC) and gallic acid conjugate *S*-(4-fluorobenzyl)-*N*-(3,4,5-trimethoxybenzoyl)-l-cysteinate (MTC). We evaluate the effects on ischemia-reperfusion-induced PC12 cells, primary neurons in neonatal rats, and cerebral ischemic neuronal damage in rats, and the results showed that MTC increased SOD, CAT, GPx activity and decreased LDH release. PI3K and p-AKT protein levels were significantly increased by activating PI3K/AKT pathway. Mitochondrial pro-apoptotic proteins Bax and Bim levels were reduced while anti-apoptotic protein Bcl-2 levels were increased. The levels of cleaved caspase-9 and cleaved caspase-3 were also reduced in the plasma. The endoplasmic reticulum stress (ERS) was decreased, which in turns the survival rate of nerve cells was increased, so that the ischemic injury of neurons was protected accordingly. MTC activated the MEK-ERK signaling pathway and promoted axonal regeneration in primary neurons of the neonatal rat. The pretreatment of MEK-ERK pathway inhibitor PD98059 and PI3K/AKT pathway inhibitor LY294002 partially attenuated the protective effect of MTC. Using a MCAO rat model indicated that MTC could reduce cerebral ischemia-reperfusion injury and decrease the expression of proinflammatory factors. The neuroprotective effect of MTC may be due to inhibition of the over-activation of the TREK-1 channel and reduction of the current density of the TREK1 channel. These results suggested that MTC has a protective effect on neuronal injury induced by ischemia reperfusion, so it may have the potential to become a new type of neuro-ischemic drug candidate.

## 1. Introduction

Cerebral infarction, also known as ischemic stroke, is a blood supply disorder caused by various reasons such as hypertension, smoking, improper diet, lack of physical exercise, diabetes and excessive drinking. It causes ischemic brain damage, and activates a series of cascade reactions in the cell, including various signaling pathways and causes irreversible brain tissue damage [1,2]. The most effective way to treat stroke today is to quickly clear it for reperfusion [3], namely ischemia reperfusion, but reperfusion after cerebral ischemia can lead to brain tissue damage and dysfunction in some cases, namely ischemia/reperfusion injury [4]. At present, the drugs for clinical treatment of cerebral ischemia include β-blockers [5], calcium antagonists [6], statins [7], RAS blockers [8], axonal regenerative ganglia Glycosyl ester, butyl benzene peptide, ROS scavenger edaravone, etc., however, the clinical effects are not satisfactory due to the limitations of their therapeutic targets [9]. The mechanism has not yet been fully elucidated, how to reduce cerebral ischemia/reperfusion injury has become a hot topic in global clinical medicine development. It is also of great theoretical and practical significance to investigate the pathology and treatment strategies of cerebral ischemia-reperfusion injury [10].

Hydrogen sulfide (H_2_S) has been recognized as the third gas signal molecule following nitric oxide (NO) and carbon monoxide (CO) [11]. In recent years, it has been found that physiological concentration of H_2_S in brain tissue is normal [12], and endogenous H_2_S is mainly derived from the desulfurization of cysteine [10]. In the central nervous system, physiological concentration of H_2_S regulates *N*-methyl-d-aspartate (NMDA) receptors in the hippocampus by promoting cAMP production, enhancing NMDA receptor-mediated neural responses [13]. Previous research indicate that H_2_S exerts neuroprotective effects in the nervous system through multiple mechanisms to protect damaged neurons or glial cells [14], for example, it can scavenge oxygen free radicals, and also improve the function of hypoxic neurons by reducing the expression of apoptotic proteins and activating anti-apoptotic proteins [15]. The results of H_2_S anti-neuronal apoptosis are mainly due to its ability to protect mitochondrial integrity, inhibit ROS-induced caspase-3 apoptosis pathway, inhibit mitochondrial apoptosis pathway [16], and weaken stroke-induced cognition obstacles [17,18,19]. Currently, propargylcysteine and its analogs such as ethylcysteine, allylcysteine, allylmercaptocysteine are all proven to be donors of hydrogen sulfide [20,21,22,23]. They release endogenous hydrogen sulfide from cysteine-β-synthase to protect against ischemic stroke [24], however, they cannot be administered in controlled doses due to their short half-life [25]. Also, they can be easily oxidized in the air due to the presence of amino group in their structure [26]. 

It is reported that gallic acid, which is found in plants such as rhubarb and big leafhopper, is a natural non-toxic polyphenolic compound [27]. It has antioxidant, anti-inflammatory, anti-bacterial, anti-free, anti-tumor and cardioprotective effects [28,29], and has neuroprotective effect in neurotoxicity of neurodegeneration and oxidative stress [30]. Although its ester analog is widely used as an antioxidant in the pharmaceutical industry, it has three phenolic hydroxyl groups which makes it oxidized due to its strong reducibility. The combination of two complementary biologically active skeletons has been widely used in drug design. Recently, researchers have attempted to integrate the chemical characteristics of gallic acid and methyl-l-leucine to produce a new gallic acid-l-leucine (GAL) conjugate [31,32]. The results indicate that GAL conjugates can be used as novel scaffold compounds for the development of new anti-inflammatory drugs [32]. Gallic acid-β-d-glucose conjugate (BGG) is a major component of Emblica officinalis medicinal plant and has a specific inhibitory effect on inflammatory diseases, especially diabetic eyes diseases [33]. Gallic acid and rivastigmine conjugate GA2 effectively prevented self-mediated Aβ aggregation as a new method against Alzheimer’s disease [34]. Previous studies in our laboratory have shown that SAC-garlic acid conjugates have good anti-inflammatory effect [35]. The above results suggest that the combination of the H_2_S donor derivative allylcysteine and gallic acid may have advantages over both neuroprotective and anti-inflammatory effects.

Therefore, in this paper, we designed and synthesized a conjugate of allylcysteine and gallic acid (MTC) to evaluate its biological activity. The results showed that MTC could increase the survival rate of nerve cells and protect neurons from ischemic injury by activating PI3K/AKT pathway, inhibit mitochondrial apoptosis signaling pathway, reduce ERS, and activate ERK-MEK signaling pathway.

## 2. Results 

### 2.1. Effect of MTC and Multitarget Compounds on Cell Viability after Ischemia-Reperfusion Induced PC12 Cell Injury

**Figure 1:**
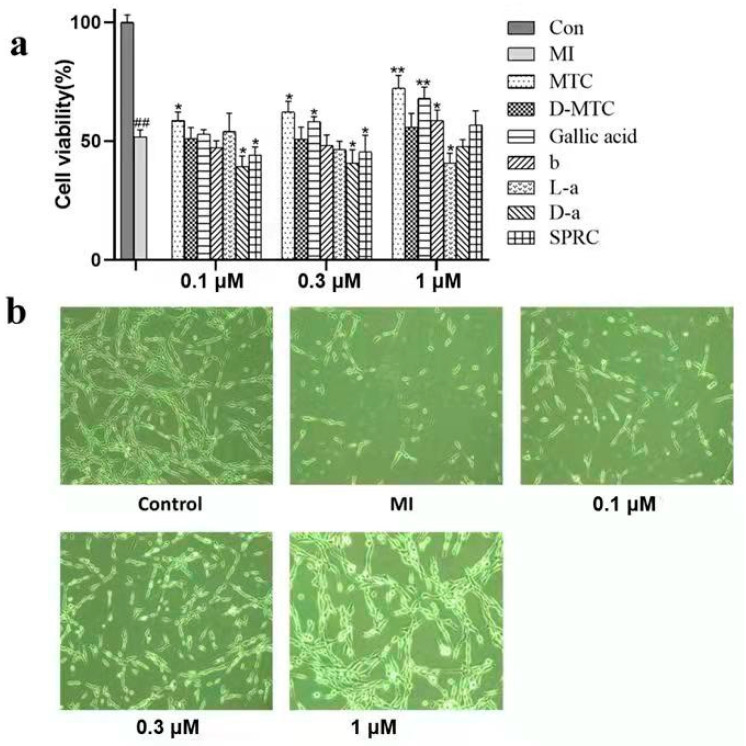
(**a**) The effects of 7 compounds treated with different concentrations (0.1 μM, 0.3 μM, 1 μM) for 24 h on cell viability after ischemia-reperfusion-induced PC12 cell injury. Which b is 3,4,5-trimethoxybenzoic acid, L-a is methyl *S*-(4-fluorobenzyl)-l-cysteinate, D-a is methyl *S*-(4-fluorobenzyl)-d-cysteinate. (**b**) Effects of MTC on PC12 injury induced by ischemia reperfusion. Data presented are mean ± SD and the figures shown are representative of three independent experiments. Compared with control group, ^##^ *p* < 0.01; Compared with myocardial infarction group (MI) group, ** *p* < 0.01, * *p* < 0.05.

The CCK-8 assay showed a significant reduction in cell viability in the ischemic group compared with the blank control group (51.81% ± 2.96%), at the same dose (0.1, 0.3, 1 μM), among the 7 compounds (MTC, D-MTC, gallic acid, 3,4,5-trimethoxybenzoic acid, methyl *S*-(4-fluorobenzyl)-l-cysteinate, methyl *S*-(4-fluorobenzyl)-d-cysteinate, SPRC), PC12 cell survival rate of MTC treatment group was higher than that of other MTC multitarget compounds treatment group, and the difference was significant compared with ischemia group (*p* < 0.05). The cell survival rate of MTC treatment group increased with the increase of concentration. The survival rate was 58.57% ± 3.72% at 0.1 μM, 62.17% ± 4.58% at 0.3 μM, and the highest number at the concentration of 1 μM (72.26% ± 3.46%). As the dose of MTC increased (Figure 1b), we chose the concentration of 1 μM as the optimal concentration. Our results indicated that MTC, a combination of allylcysteine and gallic acid, was more potent than gallic acid in neuronal repair, and we will further investigate the specific mechanism by which MTC protects neurons. The above results indicated that MTC had a concentration-dependent effect. With the increase of concentration, cell survival rate increased, cell activity was the highest when the concentration was 1 μM, which was the optimal concentration of the drug. When the concentration was 3 μM, the activity decreased, suggesting that 3 μM had certain toxicity to cells (Figure 1b).

### 2.2. MTC Post-Treatment Reduces Apoptosis Induced by Ischemia-Reperfusion in PC12 Cells

**Figure 2:**
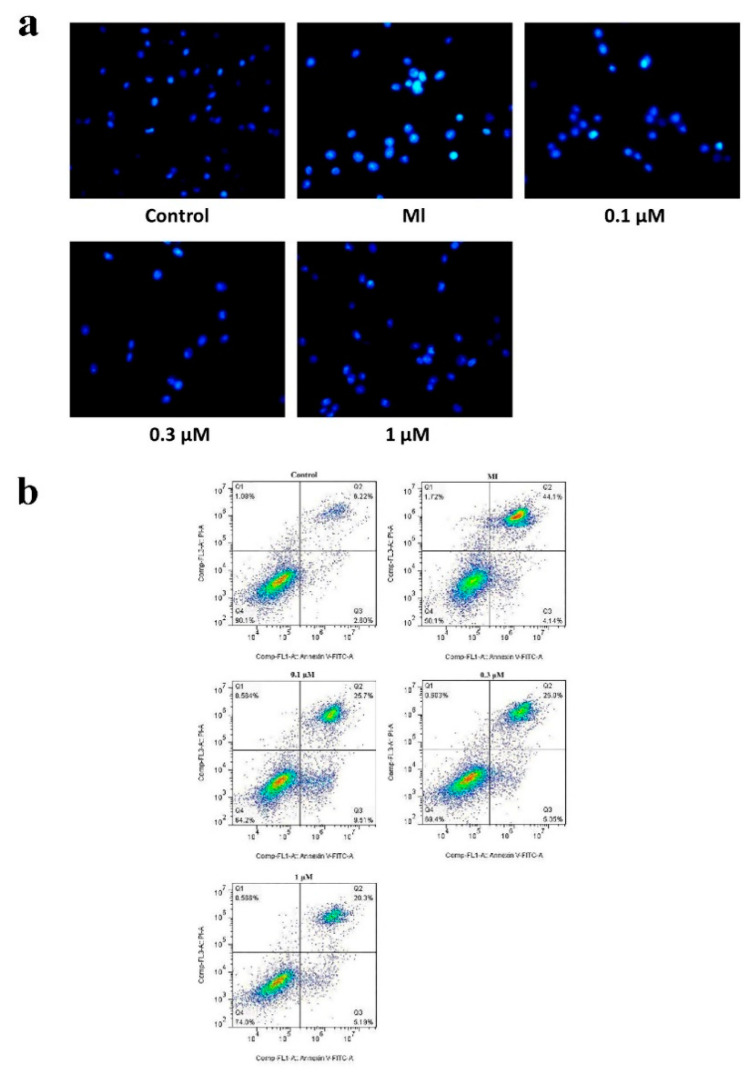
(**a**) Hoechst 33258 nuclear staining followed by fluorescence imaging was used to detect cell apoptosis. Fluorescence photomicrographs of cells stained with Hoechst 33258 (×400). (**b**) Anti-apoptotic effect of MTC on ischemic reperfusion induced PC12 injury confirmed by AnnexinV-FITC/PI.MTC was treated 24 h after ischemia.

To further validate the protective effect of MTC on PC12 cells, we next evaluated the effect of MTC on PC12 cell apoptosis. The morphological characteristics of nuclear chromatin in apoptotic cells were detected by Hoechst 33258 staining. As shown in the figure (Figure 2a), compared with the blank control group, the apoptotic cells in the ischemic group increased, and there was obvious nuclear condensation, cell membrane blistering, nuclear rupture and apoptotic bodies, showing strong blue fluorescence. After MTC (0.1, 0.3, 1 μM) treatment, the nucleus of the cell showed no obvious condensation and rupture, and showed a weak blue fluorescence with uniform dispersion. AnnexinV-FITC/PI (Figure 2b) further confirmed the anti-apoptotic effect of MTC. Compared with the blank control group, the apoptosis rate of MI (myocardial infarction) group increased. After treatment with 1 μM MTC, the PC12 cells induced by ischemia-reperfusion were significantly decreased. The results indicated that MTC achieved a protective effect by inhibiting apoptosis of PC12 cells.

### 2.3. Effects of MTC on LDH, SOD, GPx and CAT after Ischemia-Reperfusion-Induced PC12 Cell Injury

**Figure 3:**
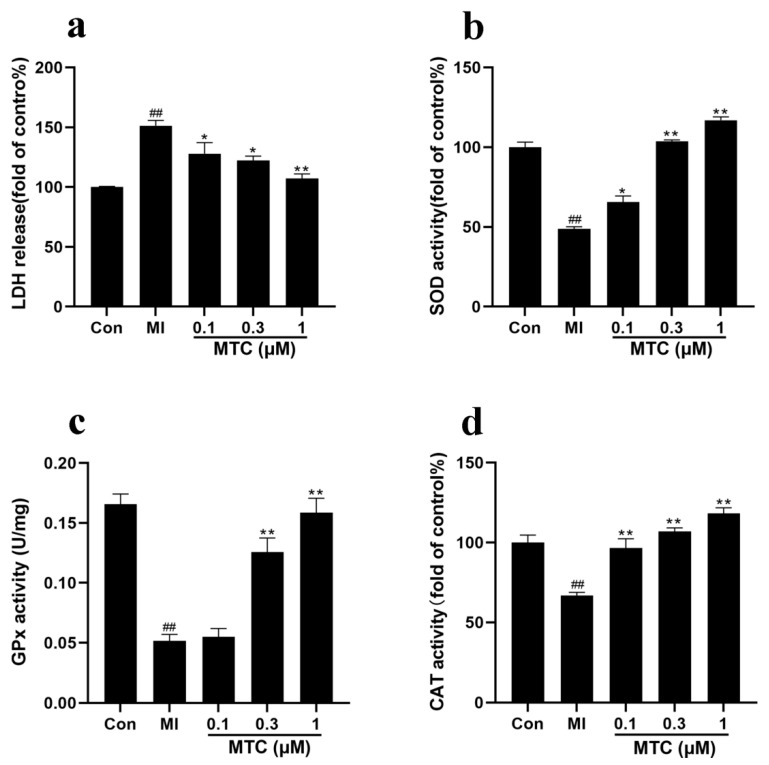
(**a**) The effect of post-treatment with *S*-(4-fluorobenzyl)-*N*-(3,4,5-trimethoxybenzoyl)-l-cysteinate (MTC) for 24 h on the release of Lactate dehydrogenase (LDH) induced by ischemia-reperfusion in PC12 cells; (**b**) The effect of post-treatment with MTC for 24 h on Superoxide dismutase (SOD) activity induced by ischemia-reperfusion in PC12 cells; (**c**) The effect of post-treatment with MTC for 24 h on GPx activity induced by ischemia-reperfusion in PC12 cells; (**d**) The effect of post-treatment with MTC for 24 h on CAT activity of PC12 cells induced by ischemia-reperfusion. Compared with control group, ^##^ *p* < 0.01; Compared with MI group, ** *p* < 0.01, * *p* < 0.05.

The results showed that (Figure 3a), the release of LDH in the dosing group was significantly lower than that in the ischemic group. When the concentration of MTC was 1 μM, the release of LDH was significantly different from that of MI (*p* < 0.01). The study showed that the activities of SOD, GPx and CAT were significantly decreased after ischemia, which was significantly different from the control group (*p* < 0.01). The activity of SOD, GPx and CAT increased after MTC treatment. The above results indicated that after MTC treatment, it had a significant enhancement of the anti-oxidative stress of PC12 cells.

### 2.4. Effects of MTC on the Levels of PI3K, p-AKT, Cleaved Caspase-9, Cleaved Caspase-3, Bax and Bcl-2 after PC12 Cell Injury Induced by Ischemia-Reperfusion

**Figure 4:**
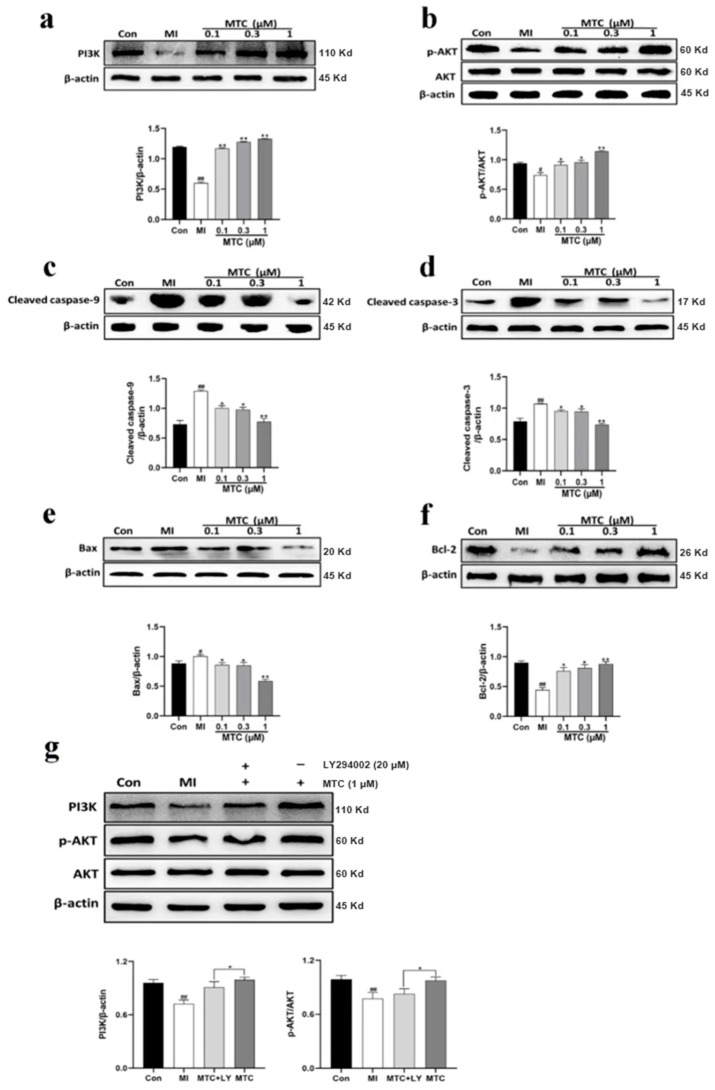
The expression of PI3K, p-AKT, cleaved caspase-9, cleaved caspase-3, Bax and Bcl-2 protein in PC12 cells was induced by ischemia-reperfusion for 24 h after MTC treatment. (**a**) Western blot analysis showed that the levels of PI3K activated by MTC post-treatment; (**b**) Western blot analysis showed that the levels of p-AKT activated by MTC post-treatment; (**c**) Western blot analysis showed that the levels of cleaved caspase-9 was inhibited by MTC post-treatment; (**d**) Western blot analysis showed that the levels of cleaved caspase-3 was inhibited by MTC post-treatment; (**e**) Western blot analysis showed that the levels of bax was inhibited by MTC post-treatment, # *p* < 0.05; (**f**) Western blot analysis showed that the levels of Bcl-2 was activated by MTC post-treatment. Compared with control group, ^##^ *p* < 0.01; Compared with MI group, ** *p* < 0.01, * *p* < 0.05; (**g**) The levels of PI3K and p-ATK showed that the treatment after MTC (1 μM) was inhibited by pretreatment with LY294002 (20 μM); Compared with control group, ^##^ *p* < 0.01; Compared with MTC + LY294002 group, * *p* < 0.05.

To further investigate the signaling mechanism of MTC on ischemia reperfusion induced PC12 cell injury, we analyzed the levels of PI3K and other proteins after treatment of PC12 cells with different concentrations of MTC (Figure 4). The levels of PI3K, p-AKT and Bcl-2 protein in the ischemic group were significantly decreased comparing with the control group (*p* < 0.01), and it was increased after MTC treatment. Compared with the control group, the levels of cleaved caspase-9, cleaved caspase-3 and Bax protein in the ischemic group were notably increased (*p* < 0.01), and it was decreased after MTC treatment. The levels of PI3K and p-AKT protein were observably decreased after pretreatment with LY294002 (*p* < 0.05), indicating that the PI3K inhibitor LY294002 inhibited the recovery of injured PC12 cells.

### 2.5. Effect of PD98059 Pretreatment on the Activity and ROS of MTC Treated PC12 Cells 

**Figure 5:**
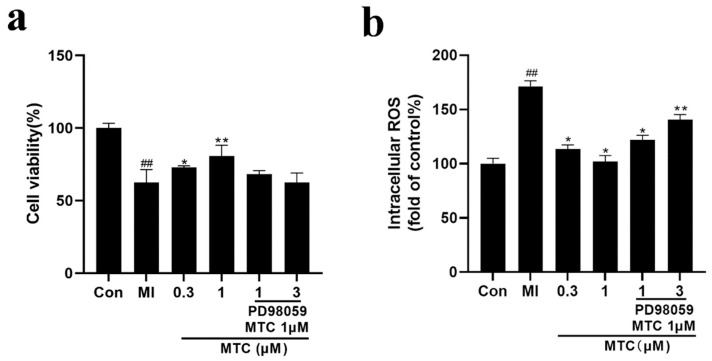
(**a**) The effects of PD98059 on the activity of PC12 cells treated with MTC; (**b**) The effects of PD98059 on MTC induced cellular ROS in PC12 cells. Compared with control group, ^##^ *p* < 0.01; Compared with MI group, ** *p* < 0.01, * *p* < 0.05.

These results indicated that MTC activates PI3K/AKT pathway to inhibit PC12 cell apoptosis, and it activates MEK-ERK pathway to promote PC12 cell proliferation. We further pretreated PC12 cells with PD98059 blocker (1 μM, 3 μM), as can be seen from Figure 5a, PD98059 pretreatment reduced cell viability, and cell viability decreased as PD98059 concentration increased. Moreover, MTC significantly inhibited intracellular ROS levels (0.3 μM, 1 μM), while PD98059 pretreatment increased cell ROS levels (*p* < 0.05 compared with 1 μM MTC group); this indicated that the MEK inhibitor PD98059 inhibited the recovery of damaged PC12 cells and impaired the antioxidant effect of MTC. 

### 2.6. Effect of MTC on Axon Growth of PC12 Cells and Neurons Induced by Ischemia-Reperfusion Injury

**Figure 6:**
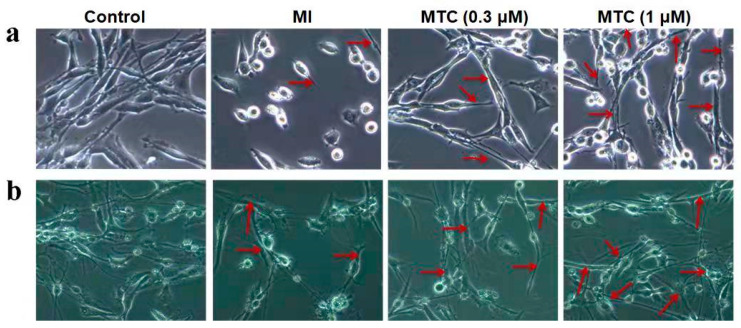
(**a**) Effects of post-treatment with MTC for 24 h on axonal growth of PC12 cells induced by ischemia-reperfusion injury; (**b**) Effects of post-treatment with MTC for 24 h on axon growth of primary neurons in rat induced by ischemia-reperfusion injury.

One of the common pathological changes in many neurodegenerative diseases and motor neuron dysfunction diseases is neuronal axon dysfunction, which ultimately leads to neuronal death [36]. More and more studies have shown that a large number of protein phosphokinases are involved in axon growth [37]. As shown in the Figure 6, the MI group inhibited axonal growth of PC12 cells and neurons, and the MTC post-treatment activated the PI3K/AKT and the MEK-ERK signaling pathways to induce axonal growth, and the number and length of axons increased significantly.

### 2.7. Effect of MTC on Expression of Endoplasmic Reticulum Stress Related Protein in PC12 Cells Induced by Ischemia-Reperfusion Injury

**Figure 7:**
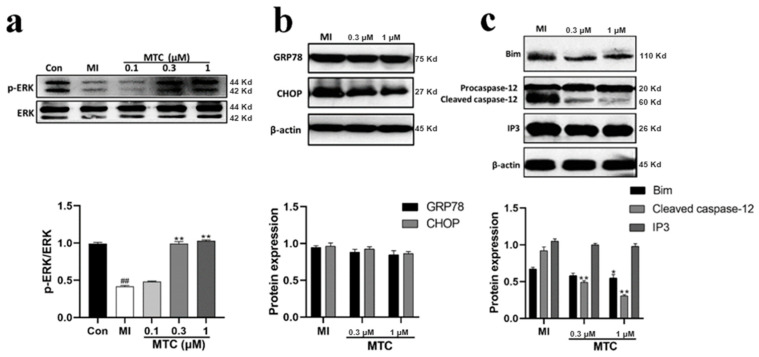
(**a**) Western blot analysis showed that MTC post-treatment activated phosphorylation of Extracellular regulated protein kinases (ERK); (**b**) Western blot analysis showed that MTC post-treatment inhibited the expression of GRP78 and C/EBP-homologous protein (CHOP); (**c**) Western blot analysis showed that MTC post-treatment inhibited the expression of Bim, caspase-12 and Inositol 1,4,5-triphosphate (IP3) proteins. Compared with control group, ^##^ *p* < 0.01; Compared with MI group, ** *p* < 0.01, * *p* < 0.05.

Up-regulation of p-ERK in the MTC post-treatment group suggested that activated AKT and MTC may activate ERK, resulting in increased cell survival and axonal regeneration after MTC treatment (Figure 7). MTC inhibited apoptosis by activating signal proteins such as AKT and Bim. Ischemia caused newly synthesized proteins to be non-glycosylated accumulating in the endoplasmic reticulum, triggering endoplasmic reticulum stress (ERS), and MTC treatment inhibited excessive ERS, thereby reducing caspase-12, GRP78 and CHOP protein levels. The above results further indicated that MTC may have a protective effect on ischemic neurons by regulating ERS.

### 2.8. MTC on Cerebral Injury Induced by Ischemia-Reperfusion in Rats

**Figure 8:**
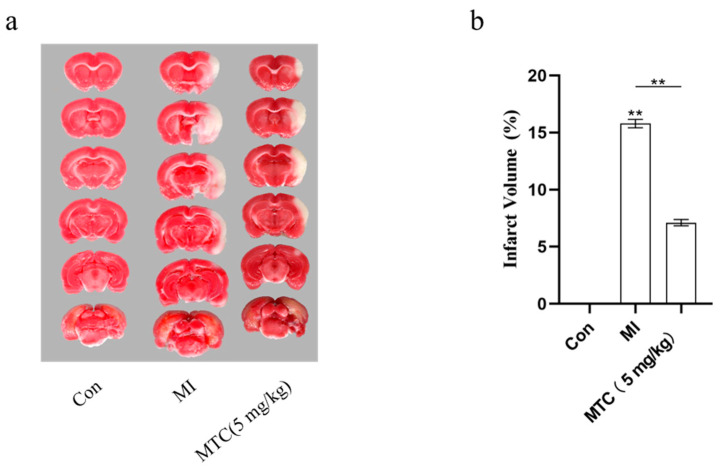
(**a**) Brain section stained with 2,3,5-triphenyltetrazolium chloride (TTC) in different groups. The white area represents the infarct part, and the red area represents the normal part. (**b**) Quantitative analysis of cerebral infarct volume, ** *p* < 0.01.

The cerebral infarct volume after ischemia-reperfusion injury was measured by 2,3,5-triphenyltetrazolium chloride (TTC) staining 24 h after the addition of MTC (Figure 8a). Almost no infarct area was observed in brain samples of the blank control group. In contrast, large infarct areas were observed in the ischemia group. Importantly, MTC decreased the cerebral infarct area in the MTC group. Data from the statistical analysis of the cerebral infarct volume provided concrete evidence that MTC administration decreased the infarct volume from approximately 50% in the ischemia group to approximately 25% in the MTC group (Figure 8b).

### 2.9. Pathological Observation of MTC on Hippocampus Injury Induced by Ischemia-Reperfusion in Rats 

**Figure 9:**
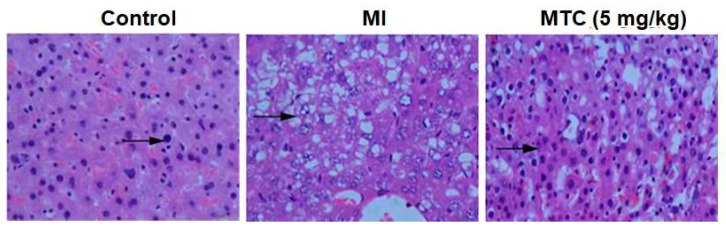
Protective effect of MTC (5 mg/kg) on injured hippocampus induced by ischemia reperfusion in rats, the black arrow point to nucleus.

Transient ischemia and hypoxia can cause severe damage in the hippocampus [9]. Based on the hippocampal tissue section of the rat ischemic model (Figure 9), it can be clearly seen that the pericellular space was widened, the apoptosis characteristics were obvious, and the number of neurons was decreased, comparing with the blank control group. Compared with ischemia group, the pericellular space was shortened and the number of neurons increased in the dosing group.

### 2.10. TREK1 Current Density and TREK1 Channel Protein Level Is Decreased by Prolonged MTC Stimulation in CHO Cells

**Figure 10:**
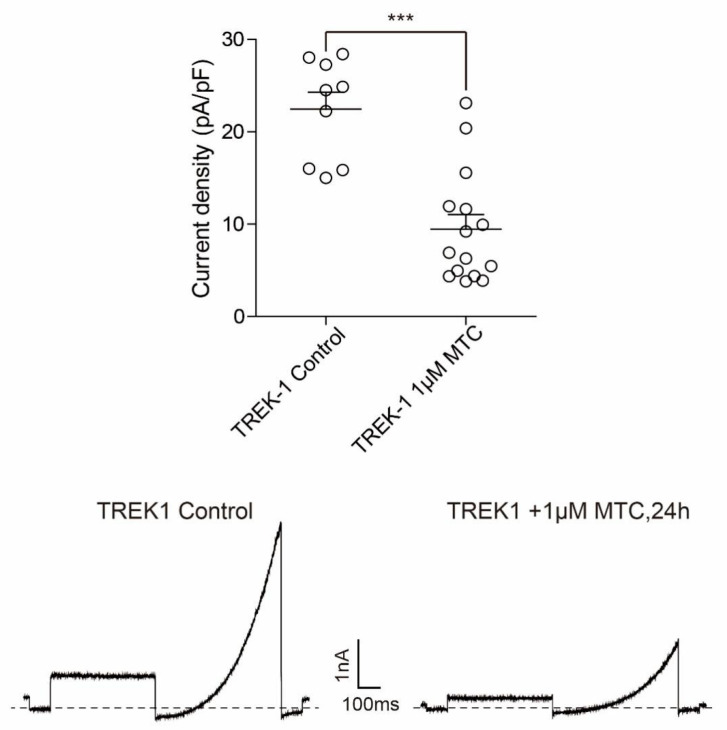
Effect of MTC (1 μM) on current density of TREK1 channel, *** *p* < 0.01.

The previously studies have shown that Potassium channels can fulfill both beneficial and detrimental roles in neuronal damage during ischemic stroke [38]. Earlier studies have characterized a neuroprotective role of the two-pore domain potassium channels KCNK2 (TREK1) and KCNK3 (TASK1): protective neuronal hyperpolarization and prevention of intracellular Ca^2+^ overload and glutamate excitotoxicity [39]. Whether the neuroprotective effect of MTC on induced cerebral ischemia in the rat middle cerebral artery occlusion (MCAO) model is due to the activation of the TREK-1 channel, and if so, whether there is a possible relationship between the mechanism of action of MTC and the TREK-1 channel, needs to be delineated further. Subsequently we investigated the changes of current density of TREK1 channel after 1 μM MTC treatment for 24 h. As shown in Figure 10, MTC can significantly reduce the current density of heterologously expressed TREK1 channel compared with the control group.

## 3. Discussion

Cerebral ischemia is the leading cause of severe disability and death, with many complications, and has become one of the major diseases that threaten human worldwide [40]. H_2_S is the third endogenous gaseous signal molecule discovered so far, which is produced in various organs and tissues and can regulate a variety of nerve functions. Low concentrations of exogenous H_2_S can protect neurons from ischemia-reperfusion injury [41]. Ischemia-reperfusion induces leukocyte activation that caused inflammation response [42], and our previous studies have shown that SAC-garlic acid conjugates have a good anti-inflammatory effect [35]. In this study, we developed a new exogenous H_2_S donor drug MTC, and evaluated the biological activity and mechanism of MTC to further explore the protective mechanism of the drug against ischemia.

H_2_S has been reported to show a dual effect in global cerebral ischemia reperfusion injury [43]. Low concentrations of H_2_S have protective effects on ischemia reperfusion injury, while high concentrations of H_2_S aggravate ischemia reperfusion brain damage and lead to toxicity. Its toxic concentration is only twice as much as endogenous hydrogen sulfide [44]. In the comparison of similar compounds, seven types of hydrogen sulfide donor compounds all can protect the ischemic nerve cells. MTC showed good effect at low concentration (1 μM) (Figure 1), while SPRC had an effect concentration of 10 μM [20]. So MTC can effectively reduce the risk of hydrogen sulfide poisoning, and the drug effect is safer and more reliable. The pathological mechanisms of neuronal death and cerebral ischemia post-perfusion injury after ischemic injury are complex, for example, the production of excitotoxic amino acid [45], destruction of calcium ion homeostasis [46], oxidative stress [47], Mitochondrial dysfunction [48], etc. ROS caused by oxidative stress will change the permeability of mitochondria, leading to mitochondrial swelling, rupture, and release cytochrome [49], which will eventually lead to necrosis or apoptosis of nerve cells. The results indicated that MTC treatment increased the SOD of PC12 cells induced by ischemia-reperfusion (Figure 3b), and reduced ROS activity (Figure 5b), exerted antioxidant effects. The activity of CAT (Figure 3d) and a GPx (Figure 3c) increase can clear peroxides away in PC12 cells, which is consistent with previous studies [50].

PI3K and its downstream molecule AKT play an important role in cell proliferation, differentiation, transcription, glucose transport, etc. [51,52,53]. This signaling pathway is activated by a variety of pathologies or emergency stimuli [54]. We demonstrated that the PI3K/AKT pathway plays a role in inhibiting apoptosis and promoting cell proliferation. The expression of PI3K and p-AKT protein levels increased (Figure 4a,b), and PC12 cell apoptosis decreased after MTC post-treatment, while the anti-apoptotic effect of MTC was inhibited by the PI3K inhibitor LY294002 (Figure 4g). Previous studies have shown that AKT can act on the proteolytic enzyme caspase-9 and inhibit its activity [55]. In this experiment, it was confirmed that activated AKT inhibits proteolytic enzyme caspase-9, thereby reducing downstream Cleaved caspase-3, thus preventing apoptosis and increasing cell survival rate. (Figure 4c,d). Under normal physiological conditions, Bcl-2 and Bax are in equilibrium. And the expression of Bcl-2 and Bax changes when cells are subjected to various pathological stimuli such as ischemia or hypoxia [56]. Our studies showed that ischemia-reperfusion increased the expression of the pro-apoptotic protein Bax and decreased the expression of the anti-apoptotic protein Bcl-2, which was consistent with previous reported results. However, MTC post-treatment increased the expression of Bcl-2 (Figure 4f), reduced the expression of Bax (Figure 4e) and inhibited apoptosis to exert its neuroprotective effect on cerebral ischemia-reperfusion injury.

The ERK pathway plays an important role in anti-apoptosis and anti-oxidative stress, and is the major kinase in the survival or apoptosis signaling pathway after neuronal injury [57]. We found that the number and length of axons increased significantly after MTC treatment of PC12 cells and primary neuronal cells (Figure 6). To figure out the mechanism of this phenomenon, we found that MTC increased the phosphorylation level of ERK protein (Figure 7a). Cell viability was inhibited after pretreatment with the MEK inhibitor PD98059, and this further confirm our view of point. Endoplasmic reticulum stress (ERS) induced by ischemia-reperfusion injury leads to neuronal apoptosis, which is characterized by elevated levels of C/EBP homologous protein (CHOP) and glucose-regulated protein-78 (GRP78), accompanied by regulating of caspase cascade protein [58]. The present study shows that MTC post-treatment reduces the expression of Bim, cleaved-caspase-12, IP3, GRP78 and CHOP protein levels (Figure 7b,c), and the results are consistent with previous studies. It was further shown that MTC activates the MEK/ERK signaling pathway to promote cell proliferation. Those proteins such as Akt and Bim which inhibit apoptosis increased by MTC, protected cells from ischemia-reperfusion injury. Ischemia leads to the inability of glycosylation of newly synthesized proteins, accumulation in the endoplasmic reticulum, triggering endoplasmic reticulum stress (ERS), excessive ERS can activate caspase-12. MTC also may impair ischemia-triggered neuronal endoplasmic reticulum stress.

Hippocampus is an important structure for studying its mechanism of neuronal injury after cerebral ischemia. However, transient ischemia and hypoxia can cause severe learning and memory impairment in the hippocampus [59]. Studies have shown that in ischemic stroke, hydrogen sulfide inhibits hippocampal neuronal damage and reduces learning and memory impairment [60]. The HE staining results (Figure 8) also showed transient ischemia and hypoxia causing severe cell death. And MTC significantly reduced hippocampal neuronal degeneration indicating that MTC can protect neurons from cerebral ischemic injury. Previous studies have shown that ischemia increased current density of TREK-1 [61,62]. Our studies showed that MTC inhibit the over-activation of the TREK-1 channel and reduce the current density of TREK1 channel, which was consistent with previous reported results.

## 4. Materials and Methods

### 4.1. Animals

Healthy adult SPF clean grade SD rats, 250–310 g, 8 weeks old, and three-day suckling mice, purchased from the Experimental Animal Center of Southern Medical University. All experimental operations strictly adhere to the “Guidance on the Treatment of Experimental Animals” (China Ministry of Science and Technology 2006) and the ethical regulations on laboratory animals of Guangzhou University and Southern Medical University.

### 4.2. Cells and Materials

Rat pheochromocytoma PC12 cells were purchased from the Chinese Academy of Sciences cell bank (Shanghai, China); fetal bovine serum and DMEM were purchased from Gibico; l-cysteine, d-cysteine, *p*-fluorobenzyl bromide, 3,4,5-trimethoxybenzoic acid was purchased from Sigma-Aldrich (St. Louis, MO, USA); anti-PI3 Kinase (CST 4255*S*), anti-AKT (CST 9272*S*), anti-p-AKT (CST 4056*S*), anti-caspase-9 (CST 9508*S*), anti-caspase-3 (CST 14220*S*), anti-bax (CST 2772*S*), anti-Bcl-2 (CST 15071*S*), anti-ERK (CST 9102L), anti-p-ERK (CST 8544*S*), anti-bim ( CST 2933*S*), anti-caspase-12 (Absin abs131292), anti-IP3 (CST 8568*S*), anti-GRP78 (Absin abs130538), anti-CHOP (CST 5554*S*), Anti-rabbit IgG (CST 7074*S*), Anti-mouse IgG (CST 7076*S*); CCK-8, LDH, SOD, GPx, CAT kits were purchased from Beyotime (Shanghai, China).

### 4.3. Chemical Synthesis

#### 4.3.1. Synthesis of *S*-(4-Fluorobenzyl)-l-Cysteine

**Scheme 1:**
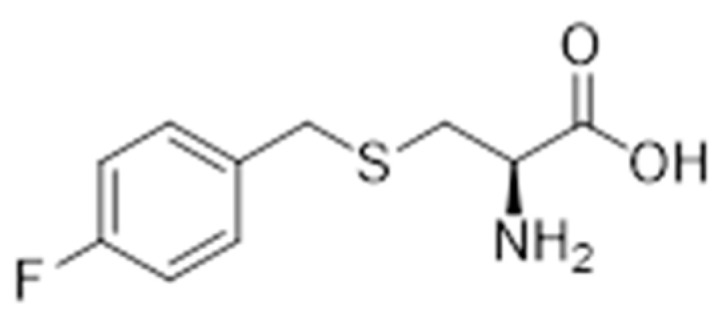
Structure of *S*-(4-fluorobenzyl)-l-cysteine.

l-cysteine (200 mg, 1.65 mmoL) was dissolved in NH_4_OH solution (2 M, 2 mL) and the mixture was stirred at 0 °C for about 30 min. Then *p*-fluorobenzyl bromide (343 mg, 1.81 mmoL) was added dropwise, stirred overnight, concentrated in vacuo and filtered. The filtrate was washed with ethanol and recrystallized from water-ethanol (1:2, *v*/*v*) to give a white solid *S*-(4-fluorobenzyl)-l-cysteine (Scheme 1).

#### 4.3.2. Synthesis of Methyl *S*-(4-Fluorobenzyl)-l-Cysteinate

**Scheme 2:**
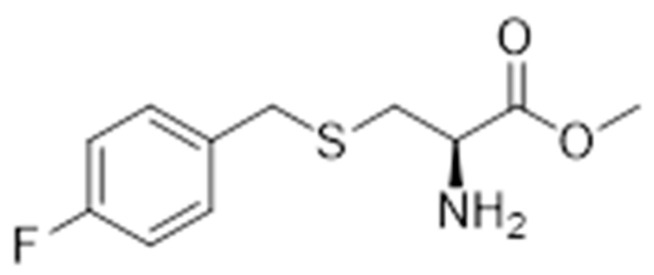
Structure of methyl *S*-(4-Fluorobenzyl)-l-cysteinate.

*S*-(4-fluorobenzyl)-l-cysteine (343 mg, 1.50 mmol) was dissolved in 8 mL of methanol, and the reaction mixture was stirred at 0 °C for about 30 minutes. Then, SOCl_2_ (713 mg, 5.99 mmoL) was added dropwise, the reaction mixture was stirred overnight, and the solvent was evaporated to give the oily compound methyl *S*-(4-fluorobenzyl)-l-cysteinate (Scheme 2).

#### 4.3.3. Synthesis of Methyl *S*-(4-Fluorobenzyl)-*N*-(3,4,5-Trimethoxybenzoyl)-l-Cysteinate

**Scheme 3:**
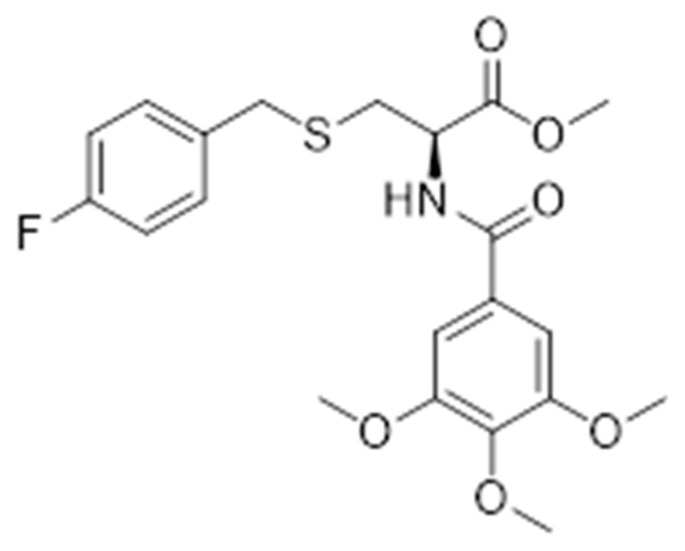
Structure of methyl *S*-(4-fluorobenzyl)-*N*-(3,4,5-trimethoxybenzoyl)-l-cysteinate.

We dissolved 3,4,5-trimethoxybenzoic acid (215 mg, 1.01 mmoL) in thionyl chloride (3 mL), refluxed for 2 hours, and the reaction progress was monitored by thin layer chromatography (TLC). The intermediate is 3,4,5-trimethoxybenzoyl chloride, which was dissolved in dichloromethane (3 mL), then triethylamine (310 mg, 3.06 mmol) was added. After stirring for 30 minutes under ice-cooling, methyl *S*-(4-fluorobenzyl)-l-cysteinate was added, and the mixture was stirred until the end of the reaction. The solvent was concentrated under reduced pressure, and the residue was applied to silica gel column chromatography to give the desired product methyl *S*-(4-fluorobenzyl)-*N*-(3,4,5-trimethoxybenzoyl)-l-cysteinate (281 mg, yield 76%) (Scheme 3).

^1^H-NMR (400 MHz, Chloroform-d) δ 7.28–7.23 (m, 2H), 7.04 (s, 2H), 6.97 (t, *J* = 7.8 Hz, 2H), 6.89 (d, *J* = 6.5 Hz, 1H), 5.02–4.95 (m, 1H), 3.91 (dd, *J* = 3.8, 2.1 Hz, 9H), 3.79 (d, *J* = 2.3 Hz, 3H), 3.72 (s, 2H), 3.06 (ddd, *J* = 14.0, 5.2, 2.0 Hz, 1H), 2.95 (ddd, *J* = 14.1, 5.9, 2.1 Hz, 1H).

^13^C-NMR (101 MHz, CDCl_3_) δ 171.4, 166.6, 163.1, 160.7, 153.2, 141.4, 133.2, 133.2, 130.4, 130.3, 128.9, 115.5, 115.3, 104.6, 60.8, 56.3, 52.7, 52.1, 35.8, 33.3.

### 4.4. Cell Culture and Transfection 

PC12 cells were cultured in high glucose DMEM medium containing 10% FBS + 2% double antibody (100 U/mL penicillin and 100 mg/mL streptomycin), and placed in a 37 °C, 5% CO_2_ incubator; every 1–2 days the solution was changed, and the cells were passaged for 2 to 3 days, and the logarithmic growth phase cells were used for the experiment. COS-7 cells were cultured in Dulbecco’s modified Eagle’s medium (DMEM) (Life Technologies, Carlsbad, CA, USA) containing 10% fetal bovine serum (FBS) and maintained at 37 °C with a 5% CO2 atmosphere. At 60–70% confluence, 3000 ng of plasmids expressing the channel cDNAs of human TREK1 (Gene ID: 3776) were cotransfected with plasmids expressing EGFP (as a marker) into COS-7 cells with a ratio of 9:1 using 10 μL of Lipofectamine 2000 (Invitrogen, Waltham, MA, USA) into 12-wellplates. Then, 24 h after transfection, the cells were split by trypsin-EDTA and replated onto 10 mm coverslips coated with 0.1 mg/mL poly-l-lysine (Sigma-Aldrich, St. Louis, MO, USA) for electrophysiology experiments.

### 4.5. Establishment and Grouping of Ischemic Models

The cells in the logarithmic growth phase were inoculated into different culture plates at 1 × 105 cells/well according to the experiment and cultured in high glucose DMEM medium containing 10% FBS + 2% double antibody for 24 h, and the old culture solution was discarded. The cells were washed once with PBS, and the cells were divided into blank control group, ischemic group, and post-ischemia dosing group (0.1, 0.3, 1, 3 μM). The blank control group was added to the normal culture medium, and the other groups were added with the same volume of ischemic solution (buffer containing 20 mM 2-DOG, 20 mM Sodium lactate, 2.5 mM Na_2_S_2_O_4_) and placed in the incubator for 20 min, the ischemic solution was discarded and wash it with PBS. One time, high-dose DMEM medium containing 10% FBS + 2% double antibody was added to each well, and the MTC was added to the final concentration of 0.1, 0.3, 1, 3 μM, respectively. After continuing to culture for 24 hours, it was used for subsequent experiments.

### 4.6. Cell Viability Assay

The cells were seeded at 1 × 10^5^ cells/well in a 96-well plate at a volume of 100 μL per well, and each group was made up to 6 replicate wells in parallel. After 24 h, 10 μL of CCK-8 solution was added to each well, and incubation was continued for 2 hours in the incubator, and the absorbance at 450 nm was measured with a microplate reader.

### 4.7. Hoechst 33258 Nuclear Staining

The cells were seeded at 1 × 10^5^ cells/well in 6-well plates at a volume of 2 mL per well. After 24 h of drug culture, the culture solution was discarded, and the cells were washed once with PBS. The cells were fixed with 4% paraformaldehyde at 4 °C for 10 min, and 1 mL of 0.5 μg/mL Hoechst 33258 staining solution was added to each well in the dark, and stained at room temperature for 25 min. Washed 3 times with PBS, observed and photographed with a magnification of 400× under a fluorescence microscope.

### 4.8. FITC-ANNEXinV/PI Dual Staining

The cells were seeded at 1 × 10^5^ cells/well in 6-well plates at a volume of 2 mL per well. After 24 h of dosing, the cells were collected by centrifugation at 1000 rpm for 5 min, the supernatant was discarded, and the cells were washed twice with pre-cooled PBS. Adjust the number of cells per tube to 0.2~1.0 × 106, and resuspend the cells by adding 400 μL of 1× Binding Buffer. The sample was sequentially added with 5 μL of FITC-AnnexinV in each tube, and reacted at room temperature (25 °C) for 15 min in the dark. Then, 10 μL of PI was added in order, and the mixture was lightly mixed, and reacted at 4 °C for 5 min in the dark. After the reaction, the machine can be used for flow analysis.

### 4.9. LDH, SOD, CAT and GPx Analysis

Cells were seeded in 96-well plates at a volume of 200 μL per well, and six replicate wells were made in parallel for each group. After incubating for 24 h, 120 μL of the supernatant was pipetted into a new 96-well plate, and LDH activity was detected using an LDH test kit. Then, the cells were seeded in a 6-well plate at a volume of 2 mL per well. After the drug was cultured for 24 h, the culture solution was discarded, and the cells were washed once with PBS at 4 °C, and then the cells were transferred to a corresponding centrifuge tube by using a cell scraper, centrifuged at 5000 rpm for 6 min, the supernatant was discarded, and 60–80 μL was added. Lysis buffer was appropriately blown to fully lyse the cells, shaken at room temperature for 30 min at a horizontal shaker, centrifuged at 13,200 rpm for 10 min, and the supernatant was taken to a new centrifuge tube. The protein concentration was determined by BCA kit, and the kit was used to detect SOD, CAT and GPx activity.

### 4.10. Western Blot Analysis

The cells were seeded at 1 × 10^5^ cells/well in 6-well plates at a volume of 2 mL per well. After the drug was cultured for 24 h, the culture solution was discarded, and the cells were washed once with PBS at 4 °C, and then the cells were transferred to a corresponding centrifuge tube by using a cell scraper, centrifuged at 5000 rpm for 6 min, the supernatant was discarded, and 60–80 μL was added. Lysis buffer was appropriately pipetted to fully lyse the cells, and shaken at room temperature for 30 min at a horizontal shaker, centrifuged at 13,200 rpm for 10 min, and the supernatant was taken to a new centrifuge tube, and the protein concentration was determined using a BCA kit. The sample was loaded with 10 μg of total protein, subjected to SDS-PAGE electrophoresis, transferred to a PVDF membrane, blocked with 5% milk for 1 h at room temperature, and applied at 4 °C overnight, and the corresponding secondary antibody was applied at room temperature for 1.5 h. The optical density of the protein bands on the membrane was analyzed by an ECL luminescent solution in a molecular imager (ChemiDoc XPS+, Bio-Rad, Hercules, CA, USA).

### 4.11. Primary Neuron Culture

After the baby rats were disinfected within three days, the ice bag was sacrificed on the neck, and the brain tissue was taken out and washed twice with 2% double-antibody PBS at 4 °C to remove the meninges and blood vessels. After cutting, the tissues were dispensed into a 10 mL centrifuge tube, centrifuged at 1000 rpm for 5 min, and the supernatant was discarded and washed once with PBS. We then added 3 mL accutase enzyme, shook the incubator at 37 °C for 10 min to obtain a tissue slurry, added the same amount of FBS-containing medium (DMEM + 10% FBS + 2% double-antibody) to terminate the digestion, and used a 200 mesh filter to remove the bulk tissue. We then centrifuged and discarded the supernatant, washed the samples once with PBS, and resuspended them in culture medium (containing 2% B27, 20 ng/mL EGF, 20 ng/mL FGF, 1% double-antibody DMEM/F12), evenly distributed the samples to a 10 mg/L polylysine-coated six-well plate, then placed it in a 37 °C 5% CO_2_ incubator. After 24 h, the culture medium was replaced. After 2–4 days of inoculation, the cells were treated with 5 μmol/L cytarabine for 24 h and used for experiments.

### 4.12. Focal Cerebral Ischemia-Reperfusion Model in Rats

The adult SD rats were randomly divided into three groups: sham operation group, model group, and MTC treatment group (5 mg/kg), four rats in each group. In the MTC treatment group, the drug was given every day for three days before the model, and 30 min after the last dose, the model was made by MCAO. Rats were anesthetized with 10% chloral hydrate at a depth of 4 mL/kg. After disinfection, a midline incision was made in the neck. The blunt dissection was performed to expose the right common carotid artery (CCA), internal carotid artery (ICA), and external carotid artery (ECA). The CCA proximal end and the ECA telecentric end were separated and ligated. At the telecentric end of the CCA ligation, a 0.26 mm suture was inserted, passed through the ICA until the middle cerebral artery (MCA) was blocked, and the skin was sutured by wire fixation. After 2 h, we pulled out the plug and reperfusion. The sham operation group did not insert the thread plug, and the other treatments were the same as the previous steps. After 24 h, the rats were sacrificed by cervical dislocation, and the brain was immediately opened for craniotomy. The tissue was fixed with 4% paraformaldehyde solution.

### 4.13. TTC Staining

The rat brains were Freeze at −20 ° C for 20 minutes, and cut into 6 sections, each of 2 mm thick. Each section was immediately stained with 1% 2,3,5-triphenyltetrazolium chloride (TTC) solution for 30 min at 37 °C and fixed in 4% Paraformaldehyde (PFA) solution. Infarct areas were unstained, and normal areas were stained red. Then each section was photographed, and the infarct areas of the brain were calculated by ImageJ. Infarct volume was calculated according to Liwei Tan’s method [63] (Equation (1)), the calculation formula is as follows:

(1) $$V=\overset{n-1}{\underset{i=1}{\sum }}\frac{A_{i}+A_{i+1}}{2}H$$

where, V is the infarct volume, Ai is the infarct area of each section, H is the section thickness.

### 4.14. HE Staining

The hippocampus of the rat brain was frozen in sections 4~8 μm, dewaxed in xylene for 5–10 min, dehydrated by 100%, 95%, 85%, 70% alcohol, and the levels were 2~5 min. Finally, it is transferred to the dyeing solution by distilled water, and the hematoxylin dyeing solution was dyed for 5 to 15 min. Excess dye solution was washed from the glass slide, using 0.5~1% hydrochloric acid alcohol (70% alcohol preparation) for color separation for a while. Microscopic examination was performed until the chromatin in the nucleus and nucleus was clear, about 10 s. Then, it was rinsed with running water for 15 to 30 min or alkalinize or blue in a short time in a saturated solution of lithium carbonate; that is, the nucleus was blue. Then, it was rinsed with distilled water, stained with 0.1~0.5% Eosin dyeing solution for 1~5 min, dehydrated by 70%, 85%, 95%, 100% alcohol, all levels were 2~3 min, excess xylene around the slice was wiped off, and to avoid drying up, the right amount of neutral gum was quickly added, and then a cover slip added to seal.

### 4.15. Whole-Cell Patch Clamp Recording

For mutagenesis, the Quick Change lI Mutagenesis kit (Agilent Technologies, Santa Clara, CA, USA) was used to introduce mutations into TREK1 channels. TREK1 Δ1-56 (the first 56 amino acids were deleted) was made by PCR as previously. All clones were verified by further sequencing of the complete open reading frame region.

Electrophysiological Recordings. Wild-type or mutant TREK1 channels were recorded 24–48 h after transfection by the whole cell patch clamp technique. Currents were measured with a MultiClamp 700B patch-clamp amplifer/Digidata 1550B digitizer and pClamp 10 software (Molecular Devices, San Jose, CA, USA). The sampling rate was 20 kHz and digitally filtered at 2 kHz. Series resistance compensation was set to 60–80%. The electrodes were pulled from borosilicate glass capillaries (BF150-110-10, Sutter Instruments, Novato, CA, USA) and had a resistance of 2–5 MΩ when filled with intracellular solution. The intracellular solution contained 140 mM KCl, 1 mM MgCl_2_, 10 mM HEPES, and 5 mM EGTA (pH adjusted to 7.4 with KOH). The extracellular solution was composed of 145 mM NaCl, 2.5 mM KCl, 1 mM CaCl_2_,3 mM MgCl2, and 10 mM HEPES (pH adjusted to 7.4 with NaOH).

### 4.16. Statistical Analysis

All data were expressed as mean ± Standard Deviation (S.D.). Difference significance was analyzed by one-way analysis of variance (ANOVA) with significant differences between groups of samples or with Origin 7.0 (OriginLab, Northampton, MA, USA) to perform Fisher’s exact probability test. Differences were considered statistically significant at *p* < 0.05.

## 5. Conclusions

In this study, we designed and synthesized a *S*-allyl-l-cysteine (SAC) and gallic acid conjugate *S*-(4-fluorobenzyl)-*N*-(3,4,5-trimethoxybenzoyl)-l-cysteinate (MTC). Compared with previous H_2_S donors, these results show that MTC has a better protective effect on ischemic neurons and requires less dosage. SOD increased and ROS decreased after MTC post-treatment to increase cellular antioxidant activity. It also makes the PI3K/AKT pathway activated, mitochondrial apoptosis signaling pathways inhibited, and endoplasmic reticulum stress reduced to increase cell viability. The results indicate that MTC has both ischemic neuron protection and ischemic anti-inflammatory effects although the effect of MTC on inflammatory factors in cerebral ischemia rats needs further study. These results provide important experimental foundation for further design and modification of conjugates of *S*-allyl-l-cysteine (SAC) and gallic acid.

## Data Availability

Not applicable.

## Sample Availability

Samples of the compounds are not available from the authors.
